# Facile Synthesis of Multi-Emission Nitrogen/Boron Co-Doped Carbon Dots from Lignin for Anti-Counterfeiting Printing

**DOI:** 10.3390/polym14142779

**Published:** 2022-07-07

**Authors:** Xuexin Gu, Lingli Zhu, Dekui Shen, Chong Li

**Affiliations:** 1 Key Laboratory of Energy Thermal Conversion and Control of Ministry of Education, School of Energy and Environment, Southeast University, Nanjing 210096, China; 220180479@seu.edu.cn (X.G.); linlyzhu@163.com (L.Z.); 2 School of Chemical Engineering, Dalian University of Technology, Dalian 116024, China; ichong612@163.com

**Keywords:** lignin, multi-emission, carbon dots, N, B co-doped, density functional theory, multilevel anti-counterfeiting

## Abstract

The transformation of lignin with natural aromatic structure into value-added carbon dots (CDs) achieves a win-win situation for low-cost production of novel nanomaterials and reasonable disposal of biomass waste. However, it remains challenging to produce multi-emission CDs from biomass for advanced applications. Herein, a green and facile approach to preparing multi-emission CDs from alkali lignin via N and B co-doping is developed. The obtained N and B co-doped CDs (NB-CDs) show multi-emission fluorescence centers at 346, 428 and 514 nm under different excitations. As the doping amount of N and B increases, the fluorescence emission band gradually shifts to 428 and 514 nm, while that at 346 nm decreases. The fluorescence mechanism is explored through the research of the structure, composition and optical performance of NB-CDs in combination with density functional theory (DFT) calculations. It demonstrates that the effect of doping with B-containing functional groups on the fluorescence emission behavior is multivariate, which may be the crucial contribution to the unique multi-emission fluorescence of CDs. The multi-emission NB-CDs with prominent stability are applied for multilevel anti-counterfeiting printing. It provides a promising direction for the sustainable and advanced application of biomass-derived CDs, and the theoretical results highlight a new insight into the deep understanding of the multi-emission fluorescence mechanism.

## 1. Introduction

The optical anti-counterfeiting technique plays an important role in financial instruments, luxury goods, classified papers, etc., owing to its flexibility of identification and difficulty in replication [1,2]. The conventionally used optical anti-counterfeiting materials have high fluorescence efficiency and excellent chemical stability but high cost and toxicity [3,4]. Carbon quantum dots (CDs), as emerging fluorescent materials with the advantages of high fluorescence efficiency, outstanding stability, low toxicity and tunable fluorescence emission, have achieved rapid development in optical anti-counterfeiting applications [5,6]. Notably, the common CD-based anti-counterfeit label with single fluorescence emission suffers from being easily forged, which cannot meet the requirement of a high-security level of anti-counterfeiting [7,8].

To address the above challenge, the multi-emission CD anti-counterfeiting materials have been developed for achieving multiple kinds of fluorescence in a single security label by changing stimuli factors, thus realizing advanced multi-level optical anti-counterfeiting [9,10,11]. Wang et al. [12] prepared dual-emission CDs with yellow/orange fluorescence from phloroglucinol dihydrate, boric acid and ethylenediamine by a one-step microwave method, which exhibited a high solid-state fluorescence QY of 39.0%. Li et al. [13] prepared dual-emission CDs through facile hydrothermal carbonization of alizarin carmine as the precursor, exhibiting attractive blue/red dual-emissive fluorescence at 430 and 642 nm. Yu et al. [9] prepared CDs from α-lipoic acid and ethylenediamine and in situ introduced them into Eu-substituted AlPO4-5 zeolite. The as-prepared composite exhibited triple-emission fluorescence of pink, blue and green color at 254 and 365 nm excitation, which were successfully applied in multilevel optical anti-counterfeiting that was hard to copy. Nevertheless, research on the multi-emission CD-based materials towards multi-level anti-counterfeiting applications is not enough. The aforementioned organic precursors used to prepare multi-emission CDs are expensive, toxic or harmful, and the complicated multi-emission fluorescence mechanism is unclear. Multi-emission CDs derived from biomass waste have the merits of low cost, environmental friendliness and renewability [14,15,16], which promote the large-scale production and commercial application of CDs in the field of anti-counterfeiting [17,18].

Herein, a green and facile two-step route is developed to synthesize multi-emission CDs using alkali lignin (AL) and 3-aminophenylboronic acid as the precursor and dopant. The doping amount of nitrogen and boron is optimized by adjusting the concentration of acid dopant. The structure, composition and optical properties of nitrogen and boron co-doped CDs (NB-CDs) are investigated to explore the complicated fluorescence mechanism in combination with density functional theory (DFT) calculations. The multi-emission CDs are later utilized as fluorescent ink for triple-level anti-counterfeiting applications. This study hopes to offer new insight on the green preparation of multi-emission CDs from lignin and promote their wide application in multilevel optical anti-counterfeiting.

## 2. Experimental Section

### 2.1. Chemicals

AL with low content of sulfur (4%) was supplied by Sigma-Aldrich (Shanghai, China). 3-aminophenylboronic acid (≥98%) was obtained from Aladdin (Shanghai, China). Phosphate buffer solutions (PBS) with the pH values of 3–11 were obtained by Yida (Quanzhou, China). Deionized water (DI) was used in the experiment. The above chemicals were used without further purification.

### 2.2. Synthetic Process

**Figure 1:**
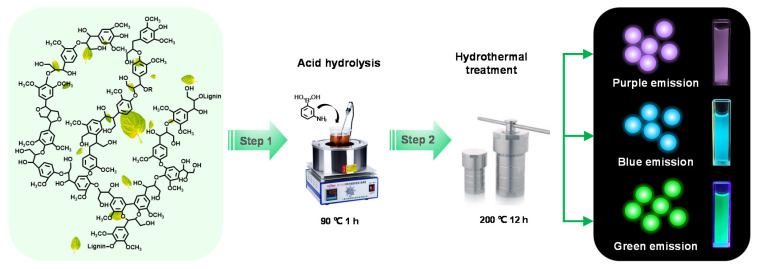
Schematic diagram of synthetic route of NB-CDs from AL and 3-aminophenylboronic acid.

The synthetic method of NB-CDs from AL and 3-aminophenylboronic acid was based on previous work [19]. The NB-CDs were synthesized by a facile two-step approach involving acidolysis and hydrothermal carbonization (Figure 1). Firstly, 0.1 g of 3-aminophenylboronic acid and 0.1 g of AL were fully dissolved into 30 mL of DI water. Afterwards, the mixed solution was subjected to heating in a water bath at 90 °C for 1 h with steady stirring at 350 rpm. The solution after the reaction was vacuum filtered by a PTFE microporous membrane of 1.0 μm. The resultant filtrate was then moved into a 50 mL Teflon-lined autoclave and maintained at 200 °C for 12 h. When naturally cooled to ambient temperature, the insoluble solid carbon in the CD aqueous solution was removed by a microporous filter membrane. The remaining supernatant was further purified in a dialysis bag (1000 Da) for 48 h. Finally, the CD solution was freeze-dried in a lyophilizer below −60 °C. For comparison, the effect of doping amount on the lignin-derived CDs was investigated by only varying the acid concentrations (0.01, 0.04, 0.07 and 0.10 mol/L). The resultant NB-CDs were obtained and denoted as CDs-1, CDs-2, CDs-3, and CDs-4.

### 2.3. Characterizations

High-resolution transmission electron microscopy (HR-TEM) was conducted using a Tecnai G2 F20 (FEI, Hillsboro, OR, USA) using an ultrathin porous carbon support membrane. The Raman spectra were measured by a DXR 2xi spectrometer (Thermo Fisher, Waltham, MA, USA). Fourier transform infrared (FT-IR) spectra were acquired on a Nicolet Is5 spectrophotometer (Thermo Fisher, Waltham, MA, USA). X-ray photoelectron spectroscopy (XPS) was carried out on a K-Alpha spectrometer (Thermo Fisher, Waltham, MA, USA). The matrix-assisted laser desorption/ionization time of flight mass spectra (MALDI-TOF MS) were performed on an ultrafleXtreme spectrometer (Bruker, Karlsruhe, Germany). Ultraviolet−visible spectra (UV-vis) were recorded on a UV-5200 spectrophotometer (Yuanxi, China). Fluorescence spectra were obtained from an Agilent Cary Eclipse. Time-resolved PL spectra were recorded using a FLS1000 spectrophotometer (Edinburgh, UK).

## 3. Results and Discussion

### 3.1. Structural Characterization of NB-CDs

**Figure 2:**
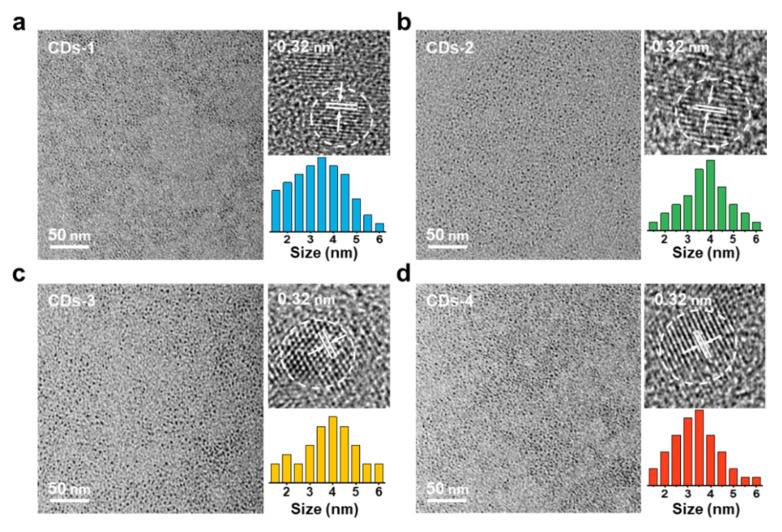
TEM, HR-TEM images and lateral size distribution diagrams of the (**a**) CDs-1, (**b**) CDs-2, (**c**) CDs-3, and (**d**) CDs-4.

**Figure 3:**
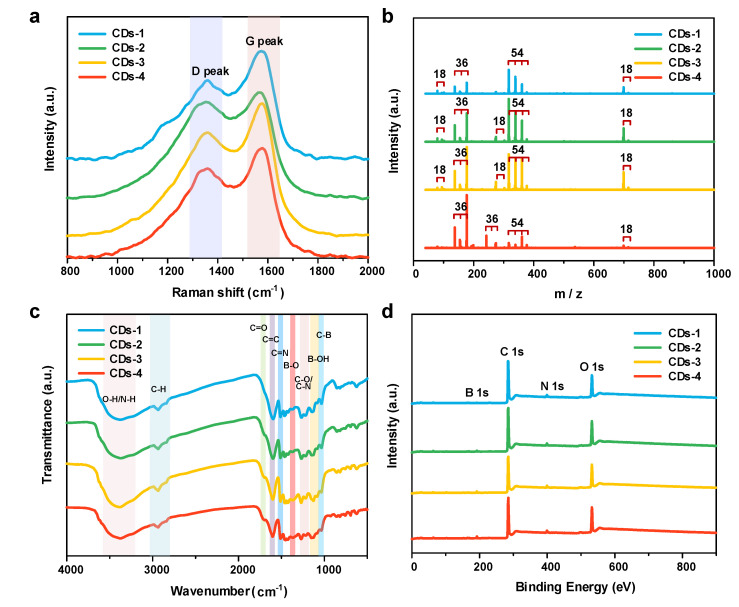
(**a**) Raman spectra, (**b**) MALDI-TOF MS spectra, (**c**) FT-IR spectra, and (**d**) XPS survey spectra of the four NB-CDs.

Four NB-CDs were prepared from lignin by adjusting the concentration of dopants via a facile synthetic approach. The micromorphology of CDs can be observed by TEM as shown in Figure 2a–d, which are all quasi-spherical nanodots. The four NB-CDs are uniformly dispersed in aqueous solution with the average lateral sizes of 3.39, 3.78, 3.82 and 3.37 nm. According to the HR-TEM images, the four NB-CDs have the same lattice fringe with a fringe spacing of 0.34 nm [20,21]. The Raman spectra of the four NB-CDs (Figure 3a) display a crystalline D band at 1356 cm^−1^ and a disordered G band at 1572 cm^−1^, representing the disordered and graphite carbon [22,23]. The intensity ratios (I_D_/I_G_) of the four NB-CDs [24,25] are 2.46, 1.68, 1.26 and 2.07, respectively. This shows that the degree of graphitization increases first and then decreases with acid concentration from 0.1 to 1.0 mol/L, indicating that small or excessive acid content is not conducive to the growth of graphitized structure in lignin-derived CDs. According to the mass spectra of the four NB-CDs in Figure 3b, their molecular weights are similar and calculated as 463.84, 469.69, 468.39, and 461.41, respectively. The multiple peaks in the mass spectra show an interval of 18n (n is an integer), which agrees well with the relative molecular mass of nH_2_O. This confirms that the conjugated graphene structure is formed in the carbon core by dehydration and condensation of the unconjugated precursor, which is consistent with the conclusions drawn from previous work [18,26].

**Figure 4:**
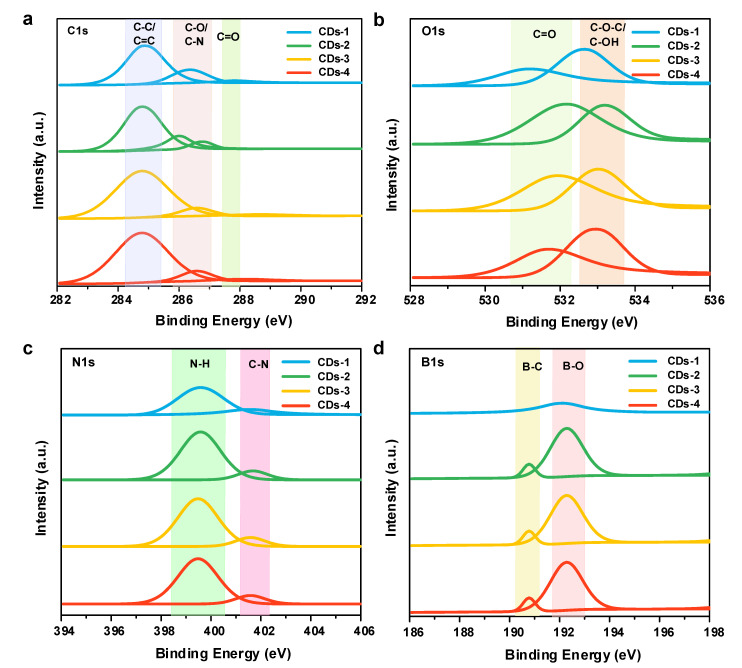
High-resolution XPS spectra of (**a**) C1s, (**b**) O1s, (**c**) N1s, and (**d**) B1s of the four NB-CDs.

To determine the surface groups and chemical composition, the FTIR and XPS spectra are characterized. As seen in the FTIR spectra (Figure 3c), the broad peaks from 3000 to 3680 cm^−1^ represent O-H/N-H functional groups. The peaks located at 2934, 1734, 1597 and 1215 cm^−1^ are assigned to the saturated C-H, C=O, C=C and C-O/C-N stretching vibrations, respectively [12]. The peaks observed at 1368, 1115 and 1033 cm^−1^ reveal the presence of B-O/B-N, B-OH and C-B functional groups [27,28]. These results suggest the successful co-doping of N and B in the lignin-derived CDs by 3-aminophenylboronic acid, which is also supported by XPS spectra. It shows that the four NB-CDs all have four prominent peaks at 284.8 eV (C1s), 532.1 eV (O1s), 190.1 eV (N1s), and 399.1 eV (B1s), respectively (Figure 3d). The corresponding deconvoluted high-resolution XPS spectra are displayed in Figure 4a–d. The C1s spectra contain three components including C-C/C=C (284.9 eV), C-O/C-N (286.3 eV) and C=O (288.3 eV). The O1s spectra are also deconvoluted into two contributions of C=O (531.9 eV) and C−O-C/C-OH (533.3 eV). The N1s spectra are the sum of N-H (399.6 eV) and C-N (401.7 eV). The B1s spectra are deconvoluted into two peaks attributed to B-C (190.6 eV) and B-O (192.3 eV) groups [12,27,28]. Furthermore, the quantitative analysis data of XPS spectra for the four NB-CDs are listed in Table S1 (see Supplementary Materials). With the increase of dopant concentration from 0.01 to 0.10 mol/L, the content of N and B doped in CDs increases first and then decreases, while the variation trend of O content is the opposite. Notably, CDs-3 possesses the optimal carbon content of 58.31% and N, B co-doping content of 4.58%.

### 3.2. Optical Performance of NB-CDs

**Figure 5:**
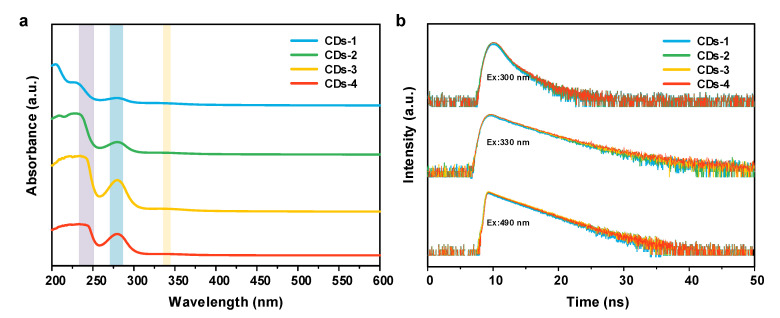
(**a**) UV-vis absorbance spectra, (**b**) time-resolved PL spectra of the four NB-CDs.

**Figure 6:**
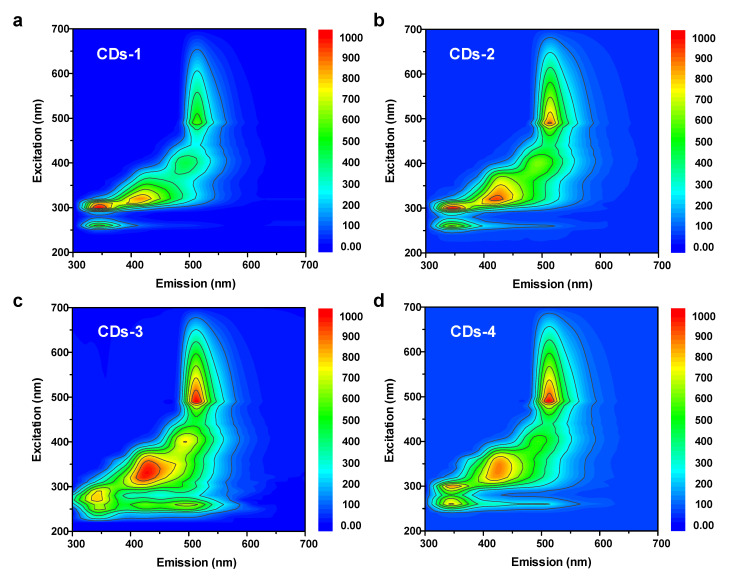
(**a**–**d**) 2D fluorescent matrix scan of the four NB-CDs.

The optical performance is presented in Figure 5a. The UV-vis spectra of the four NB-CDs all have two main absorption peaks centered at 232 and 281 nm, which correspond to the intrinsic state (π-π*) transition of C=C/C=N. The weak absorption peak located at 344 nm is assigned to the n-π* transition of C=O/C=N [26,29]. The similar absorption behaviors of four NB-CDs imply their similar structures formed via the same synthetic method. The spectrum of CD-3 with the optimal conjugated structure exhibits the strongest absorbance at 281 nm, which is also confirmed by the above XPS and Raman results. As for the PL performance of the four NB-CDs, their excitation-emission matrix spectra all exhibit a prominent excitation-dependent property (Figure 6a–d). There are three fluorescence emission centers at 346, 428 and 514 nm under the excitation of 300, 330 and 490 nm, respectively, representing purple, blue and green colors. These results demonstrate that the prepared lignin-derived NB-CDs present multi-emission fluorescence. With increasing the doping amount of nitrogen and boron, the fluorescence emission band gradually shifts to 428 and 514 nm, while that at 346 nm decreases. Notably, the spectrum of CDs-3 presents the relative long-wavelength emission and optimal fluorescence intensity, which can be evidenced by the fluorescence absolute QY values at different excitation wavelengths (Table S1). The absolute QYs of the four CDs excited at 330 and 490 nm increase in the order of CDs-1 < CDs-2 < CDs-4 < CDs-3, while the opposite is true at 300 nm. This may be associated with the difference in the chemical composition of the four NB-CDs [24,26,30].

### 3.3. Multi-Emission Fluorescence Mechanism of NB-CDs

The time-resolved PL spectra of the four NB-CDs under three excitations are exhibited in Figure 5b, which are very similar and indicate that the PL processes of all samples are similar. The decay curves are further fitted (fluorescence lifetimes are listed in Table 1), which exhibit prominent biexponential functions excited at 300 and 330 nm, while a monoexponential function is exhibited at 490 nm. The biexponential decay curves have short-lived and long-lived components (*τ*_1_ and *τ*_2_), which belong to the recombination process of intrinsic states (π-π*) and defect states (n-π*), respectively [29,31,32]. The *τ*_1_ percentages of the four NB-CDs under the excitation of 300 and 330 nm are in the ranges of 75.54–81.55% and 66.27–75.93%, respectively (Table 1). This suggests that the fluorescence emissions of purple and blue are mostly determined by the core states of NB-CDs. The percentages of *τ*_2_ at 300 nm excitation increase in the order of 2.70 ns (CDs-3) < 2.78 ns (CDs-2) < 2.84 ns (CDs-1) < 2.93 ns (CDs-4), which is consistent with the variation trend of O/C content (Table 1). It suggests that the long-lived component (*τ*_2_) excited at 300 nm may arise from the non-radiative process of C=O/C-O groups. The percentages of *τ*_2_ excited at 330 nm increase in the order of 5.56 ns (CDs-1) < 5.66 ns (CDs-2) < 6.03 ns (CDs-4) < 6.34 ns (CDs-3), which agrees well with the trend of N/C and B/C content. This implies that the long-lived components (*τ*_2_) excited at 330 nm of the four CDs are related to the N and B surface doping. Moreover, the surface defects induced by heteroatom doping have a greater impact on the PL emission behavior excited at 330 nm than that at 300 nm according to the increased percentages of *τ*_2_. The PL emissions of the four NB-CDs at 490 nm excitation all display a monoexponential decay, indicating a molecular state originated from a single fluorescence center [33,34]. The difference in origin of fluorescence emission further explains the excitation-dependent performance of NB-CDs.

**Figure 7:**
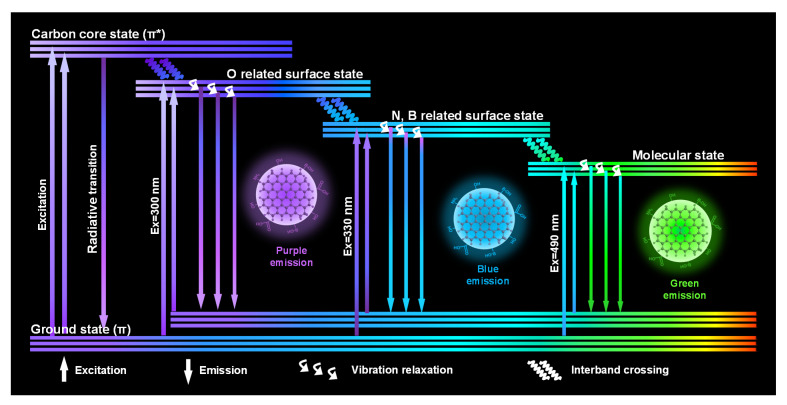
Proposed fluorescence mechanism of multi-emission NB-CDs.

The quantitative results above conduce to reveal the complicated fluorescence mechanism of NB-CDs currently. As illustrated in Figure 7, the multi-emission behavior of NB-CDs depends on the comprehensive effect of carbon core and surface defect state. In this work, heteroatom doping (O, N and B-related functional groups) is inclined to create more defective state related energy levels, which enable trapping of photoexcited electrons [12,35]. The N and B atoms, compared with the O atom, can easily replace and combine strongly with the C atom due to their similar sizes. The n electrons of N and B facilitate the transition to the internal sp2 carbon core, resulting in a reduced energy band gap and a red-shifted emission peak [36,37,38]. The various energy levels lead to several radiation recombination routes returning to the ground state, thus enhancing the surface-state-related fluorescence intensity [24,39]. The efficient separation of electrons and holes within the n-π* gap can be adjusted by varying the content of O, N, and B doping [12,27,40,41]. As proof, the variation trend of absolute QYs excited at 300, 330, and 490 nm agrees well with the doping content O, N/B, and total heteroatoms in the four NB-CDs, respectively (Table S1).

**Figure 8:**
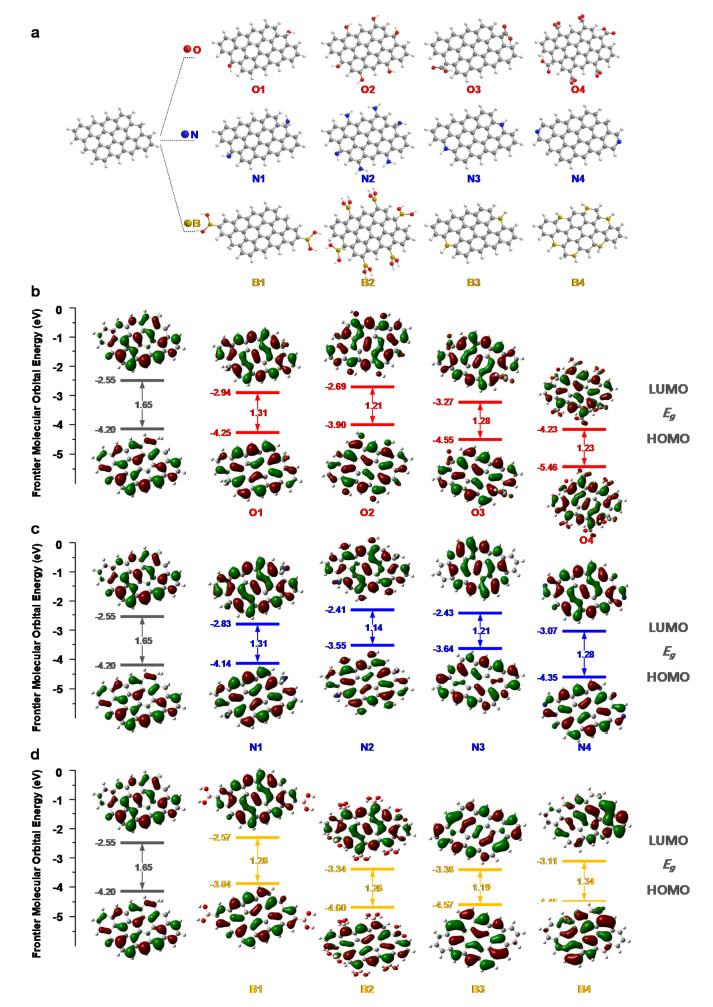
DFT calculation results. (**a**) CDs structure models and energy levels of the frontier molecular orbitals of (**b**) O-containing group, (**c**) N-containing group, and (**d**) B-containing group in combination with 12 benzene rings.

The complicated fluorescence mechanism of NB-CDs is further explored in depth by DFT calculations. A series of CD molecules composed of 12 aromatic rings fused with heterogeneous functional groups are designed to investigate the surface-defect state (Figure 8a). The highest occupied molecular orbital (HOMO), lowest unoccupied molecular orbital (LUMO) energy levels and energy bandgaps (e.g.,) are calculated according to the method in the Supporting Information. As shown in Figure 8b, the HOMO and LUMO energy gradually increase and the bandgap narrows from 1.65 to 1.21 eV as the number of hydroxyl groups (–OH) increases from zero to six, while the HOMO and LUMO energy decrease in the presence of carboxyl groups (–COOH). According to Figure 8c, the bandgap decreases from 1.65 to 1.14 eV as the number of amine groups (–NH_2_) increases from zero to six. The bandgap also decreases with the addition of C-N and C=N groups. The introduced boron-containing groups lead to the reduction of the bandgap of CDs (Figure 8d). The Eg of CDs exhibits little change as the number of boron hydroxyl groups (–B–OH) increases, while the energy band gap gradually picks up as the number of carbon-boron groups (–C–B) increases. It demonstrates that the effect of doping with boron-containing functional groups on the fluorescence emission behavior of CDs is multivariate, which may be the crucial contribution to the unique multi-fluorescence emissions of CDs. The results of this theoretical calculation have been verified by previous experimental work [12,27]. The quantitative results above conduce to reveal the ambiguous fluorescence mechanism of CDs illustrated in this work.

### 3.4. Multilevel Anti-Counterfeiting Printing of NB-CDs

The NB-CDs derived from lignin with the intriguing merit of triple-fluorescence emission show great promise in advanced multi-level anti-counterfeiting applications. Besides, the fluorescence stability of CD material is known as a prerequisite for practical optical applications [9,10]. As seen in Figure S1a–c, the CDs-3 selected as a typical material is subjected to different treatments for the stability test. The PL emission spectra of CDs-3 excited at 300, 330 and 490 nm exhibit almost negligible variation. Their PL intensities can be maintained above 90% whether CDs-3 aqueous solution is stored for over 2 months or at the heating temperature up to 300 °C. The above-mentioned outstanding long-term and thermal-stability show that CDs-3-based fluorescent ink can satisfy the needs of anti-counterfeiting printing. In addition, CDs-3 is dispersed in phosphate buffer solutions (pH: 3–11) to investigate its pH sensibility. The PL intensities show small fluctuations at pH 5–9 while they decrease significantly at pH 3, 10 and 11 due to the protonation effect of –OH and –NH_2_ functional groups on CDs-3 under the extreme acidic or alkaline environment [1,22,42]. Overall, CDs-3 has high fluorescence stability, which is beneficial to the dependability of practical anti-counterfeiting applications.

**Figure 9:**
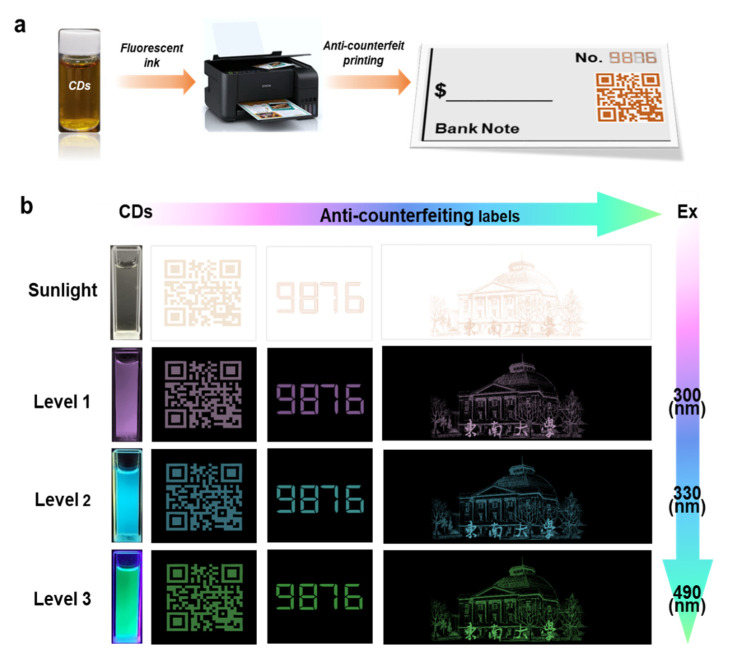
(**a**) Strategy for optical anti-counterfeiting printing using CDs-3 solution as fluorescent ink. (**b**) Photographs of triple-level fluorescence anti-counterfeiting labels in a banknote under sunlight and UV light.

The CDs-3 aqueous solution is employed as fluorescent ink for anti-counterfeiting printing, which is conducted on a desktop inkjet printer (Figure 9a). The anti-counterfeiting labels of numerical codes and QR codes are successfully printed on a banknote (made of non-fluorescent paper). These patterns printed with CDs-3 ink are nearly indistinguishable by the naked eye under sunlight, but show purple fluorescence under 300 nm UV light, blue under 330 nm UV light, as well as green after switching to 490 nm excitation (Figure 9b). Multi-peak response signals enable the efficient avoidance of interference by irrelevant factors. Remarkably, the tunable multi-emission emissions of CDs-3-based fluorescent ink realize multi-level optical anti-counterfeiting. This triple-level anti-counterfeiting process only needs one kind of stimuli factor (excitation lights with 300, 330 and 490 nm) on a single label. Compared with other complicated stimuli factors (temperature, chemical reagents, and mechanical force) [7,31,43], this is a simple, fast, and advanced triple-level optical anti-counterfeiting strategy with high security. The multi-emission biomass-derived CDs exhibit the advantages of low cost, green preparation, and high stability, and are expected to become an ideal material for future optical anti-counterfeiting applications [4,34].

## 4. Conclusions

A green and facile method has been demonstrated to prepare NB-CDs from alkali lignin and 3-aminophenylboronic acid, which exhibit a triple fluorescence emission center of purple, blue and green color under different excitation wavelengths. With increasing the doping amount of nitrogen and boron, the fluorescence emission band gradually shifts to 428 and 514 nm, while that at 346 nm decreases. According to the structure, composition, and optical properties and DFT calculations, the multi-emission fluorescence mechanism of NB-CDs is ascribed to the synergistic effect of the core state and the surface-defect state of O, N, and B. Among them, doping with B-containing functional groups may be the crucial contribution to the unique multi-fluorescence emissions of NB-CDs. The lignin-derived multiple-emission CDs can be regarded as the next generation of multi-level anti-counterfeiting materials due to their outstanding characteristics of excellent fluorescence stability, high cost-efficiency and renewability.

## Figures and Tables

**Table 1:** Fluorescence lifetimes of the four NB-CDs excited at different wavelengths.

Samples	*τ*_1_ (ns)	B_1_ (%)	*τ*_2_ (ns)	B_2_ (%)	*τ*_avg_ (ns)	χ^2^
Ex: 300 nm; Em: 346 nm						
CDs-1	0.78	75.54	2.84	24.46	1.28	0.984
CDs-2	0.86	79.61	2.78	20.39	1.25	1.064
CDs-3	0.93	81.55	2.70	18.45	1.26	1.233
CDs-4	0.90	80.87	2.93	19.13	1.29	0.996
Ex: 330 nm; Em: 428 nm						
CDs-1	2.94	68.82	5.56	31.18	3.76	0.920
CDs-2	2.99	66.27	5.66	33.73	3.88	1.110
CDs-3	3.13	75.93	6.34	24.07	3.91	0.955
CDs-4	3.10	73.66	6.03	26.31	3.87	0.973
Ex: 490 nm; Em: 514 nm						
CDs-1	3.27	100	/	/	3.27	1.054
CDs-2	3.24	100	/	/	3.24	0.978
CDs-3	3.41	100	/	/	3.41	1.041
CDs-4	3.38	100	/	/	3.38	0.997

χ refers to the confidence factor.

## Data Availability

Not applicable.

## Supplementary Materials

The following supporting information can be downloaded at: https://www.mdpi.com/article/10.3390/polym14142779/s1, Table S1. The quantitative analysis results of XPS; Figure S1. Stability test of CDs-3. Variation of PL intensity of CDs-3 aqueous solution (a) stored at room temperature from 0 to 63 days; (b) heated at different temperatures from 0 to 300 °C for 30 min, and (c) dispersed at different pH values from 3 to 11. See [44,45,46].
