# Supplementary material for: Facile Synthesis of Multi-Emission Nitrogen/Boron Co-Doped Carbon Dots from Lignin for Anti-Counterfeiting Printing
Source: Polymers (Basel). 2022 Jul 7;14(14):2779. doi: 10.3390/polym14142779 (PMC9316793; doi:10.3390/polym14142779)
Supplement: Supplementary file 1 [file polymers-14-02779-s001.zip › polymers-1799495-supplementary.pdf]

polymers

MDPI

Supporting Materials: Facile Synthesis of Multi-Emission Nitrogen/Boron Co-doped Carbon Dots from Lignin for Anti-Counterfeiting Printing

Xuexin Gu, Lingli Zhu, Dekui Shen and Chong Li

Calculation of Fluorescence Lifetime

The time-resolved photoluminescence (TRPL) decay curves are fitted by a multiexponential function R(t) (eq. (1)) and the average lifetime (τ_{avg}) can be calculated according to eq. (2).[1, 2]

$$R(\mathrm{t}) = B_1 e^{(-t/\tau_1)} + B_2 e^{(-t/\tau_2)} \ldots\ldots + B_n e^{(-t/\tau_n)} \quad (1)$$

$$\tau_{avg} = (B_1 \tau_1{}^2 + B_2 \tau_2{}^2 + \cdots .. B_n{}^2 \ \tau_n{}^2) \ / \ (B_1 \tau_1 + B_2 \tau_2 \ldots\ldots .. + B_n \tau_n) \quad (2)$$

Here, $\tau 1$, $\tau 2$ ...... τn represent fluorescence lifetimes and $B1$, $B2$ Bn represent percentages of $\tau 1$, $\tau 2$ τn, respectively.

DFT Calculation

All the calculations are carried out using Gaussian 09 suite of program. The ground-state geometries of GQDs were firstly optimized by DFT2-3 B3LYP (Becke's three-parameter hybrid function with the non-local correlation of Lee-Yang-Parr5) method with the Pople 6-31G(d) basis set (B3LYP/6-31G(d)).[3] The dangling carbon bonds are passivated by hydrogen atoms. The molecular orbits of possible moieties were imported at the B3LYP/6-31 G (d, p) level based on optimized ground-state geometries.[4, 5] Multiwfn is used for post-processing of computed resulting images.[6]

Table S1. The quantitative analysis results of XPS.

Sample	C(%)	O(%)	N(%)	B(%)	O/C(%)	N/C(%)	B/C(%)	QY1(%)	QY2(%)	QY3(%)
CDs-1	57.60	41.33	0.93	0.14	0.72	0.02	0.00	0.36	0.23	0.17
CDs-2	57.21	40.53	1.91	0.35	0.71	0.03	0.01	0.21	2.92	0.75
CDs-3	58.31	37.11	3.82	0.76	0.64	0.07	0.01	0.10	7.40	4.66
CDs-4	55.67	41.06	2.84	0.43	0.74	0.05	0.01	0.22	3.21	3.89

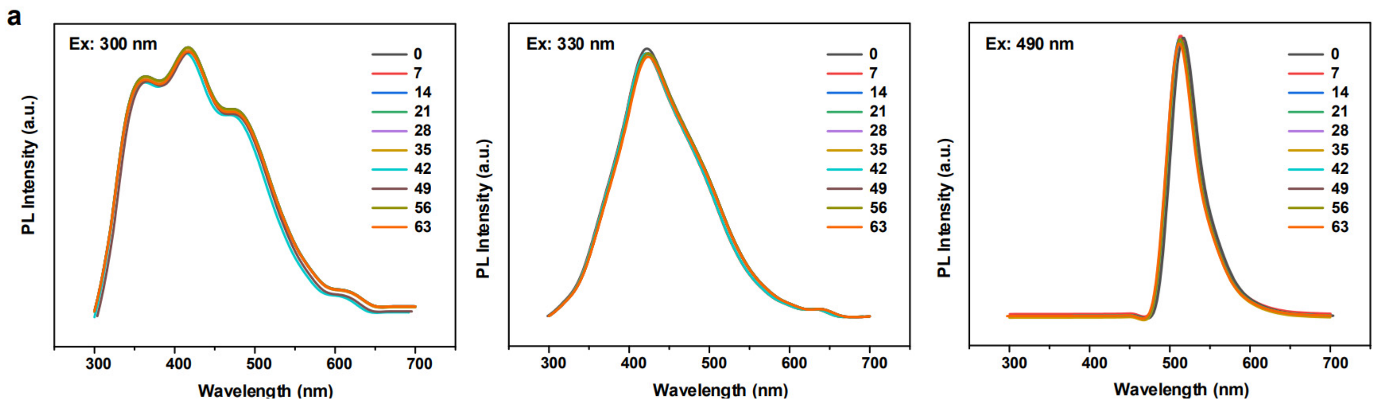

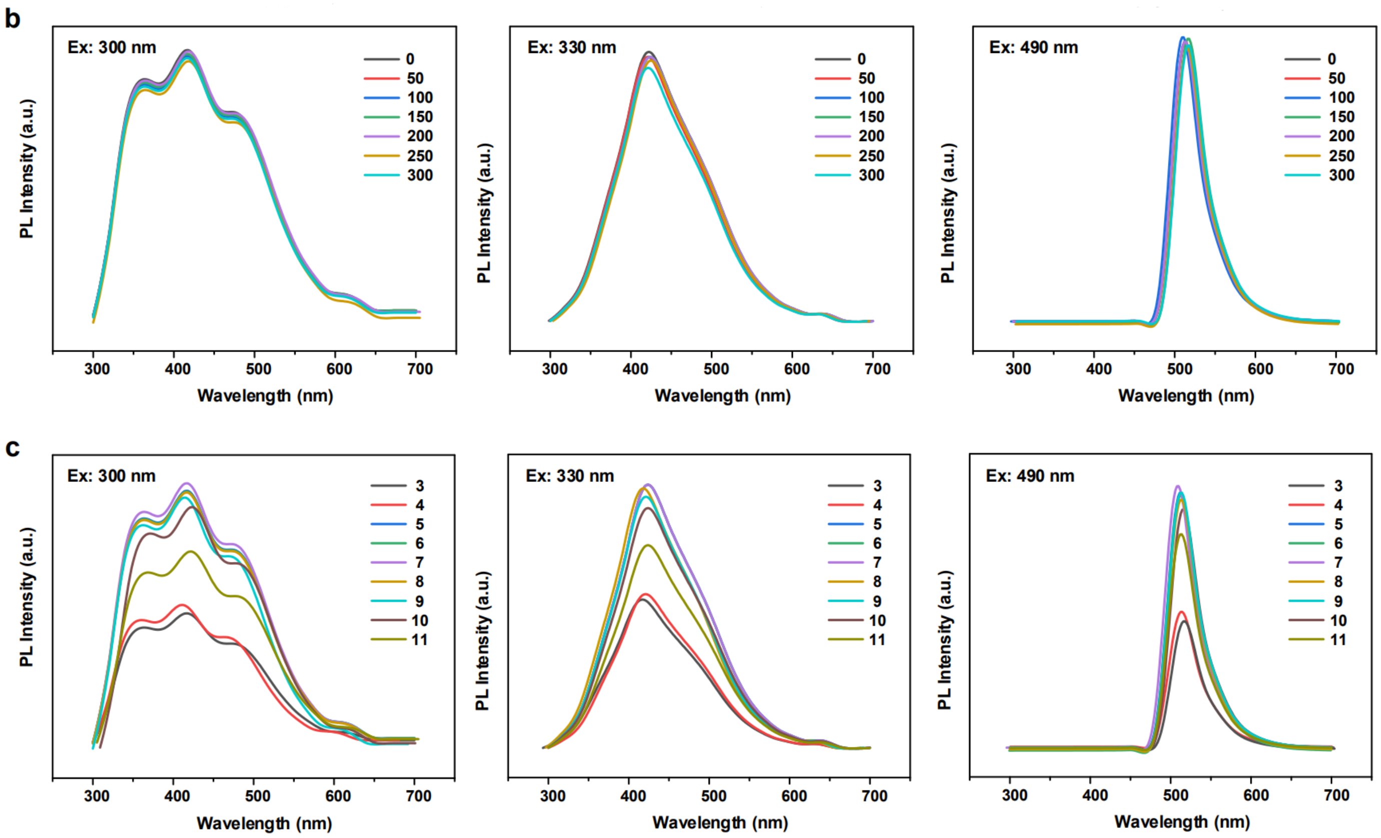

Figure S1. Stability test of CDs-3. Variation of PL intensity of CDs-3 aqueous solution (**a**) stored at room temperature from 0 to 63 days, (**b**) heated at different temperatures from 0 to 300 °C for 30 min, and (**c**) dispersed at different pH values from 3 to 11.
